# 4Ds: Documenting delirium diagnosis in discharge summary

**DOI:** 10.1192/j.eurpsy.2021.1336

**Published:** 2021-08-13

**Authors:** Z. Azvee, I. Khair, N. Barry, J. Sheehan

**Affiliations:** Liaison Psychiatry, Mater Hospital, Dublin, Ireland

**Keywords:** Patient safety, Service improvement, communication, delirium

## Abstract

**Introduction:**

Hospital discharge is a significant transitional phase with varying levels of needs and risks to be managed as lapses in communication commonly happen between secondary/tertiary and primary care.

**Objectives:**

Our aim was to look at inclusion of delirium diagnosis in discharge summaries based on standards set by: 1. Health Information and Quality Authority (HIQA) National Standard for Patient Discharge Summary Information 2. NICE Guidelines on Delirium: prevention, diagnosis and management (CG 103)

**Methods:**

All inpatients referred to Liaison Psychiatry from 9^th^July 2019 till 5^th^ January 2020 were included, n = 729. Compared discharge summaries diagnoses to the internal Liaison Psychiatry ICD 10 consensus diagnosis and also HIPE coded diagnosis specifically for delirium.

**Results:**

Delirium diagnoses and inclusion of delirium-specific information on discharge summary

**Table:** 

	n	Proportion (n=112*) (%)
Q1 Any F05 diagnosis coded by Liaison Psychiatry	117	100
Q2 F10.4 diagnosis coded by Liaison Psychiatry	0	0
Q3 F1x.4 diagnosis coded by Liaison Psychiatry	0	0
Q4 Any F05, F10.4 and F1x.4 diagnosis coded in discharge summary on patient centre	23	20.5
Q5 Was the word delirium or its synonym such as acute confusional state mentioned in the body of the discharge summary?	62	55.4
HIPE Code Diagnosis	66	58.9

**Conclusions:**

Hospital discharge summaries are essentially the main communication link between hospitalists and general practitioners to ensure continuity and future care of patients. Delirium diagnosis is not always recorded in discharge summaries. This is a risk to be managed. Education is vital to ensure awareness, prevention, early recognition and to ensure recording of diagnosis of delirium.

